# Distance from the external occipital protuberance to the occipital artery for occipital nerve block

**DOI:** 10.1007/s00540-013-1587-7

**Published:** 2013-03-11

**Authors:** Takero Arai, Kazuyoshi Ishikawa, Tomoyuki Saito, Yuichi Hashimoto, Takashi Asai, Yasuhisa Okuda

**Affiliations:** Department of Anesthesiology, Dokkyo Medical University, Koshigaya Hospital, 2-1-50, Minami-Koshigaya, Koshigaya, Saitama 343-8555 Japan

To the Editor:

Greater occipital nerve block is indicated for cervicogenic or cluster headache. Identifying the location of the nerve is important for differential diagnosis of cervicogenic headache, because it is necessary to block this nerve and not neighboring structures [1]. The blocking point is where the greater occipital nerve crosses the superior nuchal line approximately halfway between the external occipital protuberance and the mastoid process [2, 3]. The nerve emerges on this line in association with the occipital artery, and thus this artery is the most reliable anatomical landmark [2, 3]. However, it may be difficult to detect pulsation of the occipital artery, because the artery is thin, its course is variable, and hair may make palpation of the scalp difficult. Although the use of sonography has been suggested for detection of the nerve [4], it is likely to be difficult because of interference from the hair of the head. In such a case, textbooks generally describe that the blocking point should be 2.5–3.0 cm from the external occipital protuberance [2, 3]. In our daily clinical practice, we thought that this distance may be too short in men. Therefore, we formally studied this by pulsation, and by a Doppler flowmeter, which we previously reported to be useful [5].

The institutional review board approved the study, and written informed consent was obtained from 330 healthy volunteers (200 men and 130 women). Each volunteer was placed in the prone position and attempts were made to palpate the bilateral occipital arteries. If neither side could be palpated, it was judged a failure. The Doppler flowmeter probe (Nihon Kohden ES-1000 SP, Tokyo, Japan) was then placed and moved along the superior nuchal line until artery flow became audible. When the artery was detected, we measured the distance between the artery and the external protuberance.

The artery could not be palpated on either the right or the left side in 15 of 200 (7.5 %) male subjects and 12 of 130 (9.2 %) female subjects. With the Doppler flowmeter, the artery could not be detected in 5 (2.5 %) men and 3 (2.3 %) women, and in all of whom the artery also could not be detected by palpation. The mean distance from the external occipital protuberance to the occipital artery in females (Table 1) was within the textbook range (2.5–3.0 cm), but the distance in males was significantly longer than that in females (*P* ≤ 0.05, Student’s *t* test) (Table 1), and the distance was between 2.5 and 3.0 cm in only 27 of 195 (13.8 %) volunteers (in whom the distance could be measured).

**Table 1:** Volunteer characteristics and distance between the occipital artery and the external occipital protuberance (mean (standard deviation), [range])

	Males (*n* = 200)	Females (*n* = 130)
Age (years)	29 (6)	27 (6)
Height (cm)	170 (5.3)	161 (6.1)
Weight (kg)	67 (9.0)	59 (5.0)
Distance		
Right	4.1 (1.0) [2.4–6.5]*	2.8 (0.5) [2.0–4.2]
Left	3.9 (0.9) [2.0–6.0]*	2.7 (0.4) [2.0–4.1]

* *P* < 0.05 versus females

We have confirmed that the occipital artery sometimes cannot be detected by palpation, or even by Doppler flowmeter. The volunteers we studied were all young, and it is possible that the failure rate of detection may be higher in senior people with headache, who may have neuralgia-induced vasoconstriction.

We suggest that, when the occipital artery could not be detected, the blocking point should be about 2.5 cm from the external occipital protuberance in women and about 4.0 cm (and not 2.5–3.0 cm) in men.
